# Prevalence of Respiratory Syncytial Virus Infection in Hospitalized COPD Patients in Spain Between 2018–2022

**DOI:** 10.3390/diseases13010023

**Published:** 2025-01-20

**Authors:** Rosa María Gómez-García, Javier De-Miguel-Díez, Ana López-de-Andrés, Valentín Hernández-Barrera, Ana Jimenez-Sierra, Natividad Cuadrado-Corrales, José J. Zamorano-León, David Carabantes-Alarcón, Andrés Bodas-Pinedo, Rodrigo Jiménez-García

**Affiliations:** 1 Respiratory Care Department, Hospital General Universitario Gregorio Marañón, Universidad Complutense de Madrid, Instituto de Investigación Sanitaria Gregorio Marañón (IiSGM), 28007 Madrid, Spain; rosamg07@ucm.es; 2 Department of Public Health & Maternal and Child Health, Faculty of Pharmacy, Universidad Complutense de Madrid, 28040 Madrid, Spain; anailo04@ucm.es; 3 Preventive Medicine and Public Health Teaching and Research Unit, Health Sciences Faculty, Rey Juan Carlos University, 28922 Alcorcón, Spain; valentin.hernandez@urjc.es; 4 Faculty of Medicine, Universidad San Pablo Ceu, 28668 Madrid, Spain; a.jimenez100@usp.ceu.es; 5 Department of Public Health & Maternal and Child Health, Faculty of Medicine, Universidad Complutense de Madrid, 28040 Madrid, Spain; mariancu@ucm.es (N.C.-C.); josejzam@ucm.es (J.J.Z.-L.); dcaraban@ucm.es (D.C.-A.); abodas@ucm.es (A.B.-P.); rodrijim@ucm.es (R.J.-G.)

**Keywords:** COPD, respiratory syncytial virus, hospitalizations, mortality, Spain

## Abstract

Background: Respiratory syncytial virus (RSV) infection is a common cause of hospital admission. The association between chronic obstructive pulmonary disease (COPD) exacerbation and RSV infection is not well studied. Objective: To analyze the hospitalizations of patients with COPD and RSV infection in Spain between 2018 and 2022. Methods: The data used were obtained from the Spanish Hospital Discharge Database. We selected subjects aged ≥40 years diagnosed with COPD, admitted to the hospital from 1 January 2018 to 31 December 2022. The COPD population that met the selection criteria was subdivided based on the presence of an ICD-10 code for RSV infection. To obtain comparable populations, for each subject with COPD and RSV infection, a subject without an RSV code was selected, with the COPD code in the same diagnostic position (1 to 20), as well as the same year of admission, sex, and age. Results: Among subjects aged ≥40 years, 1,429,288 were identified as having COPD, of whom 5673 also had RSV infection. The number of hospitalizations with COPD and RSV infection increased during the study period. The proportion of RSV infection among patients admitted for COPD increased significantly over time, from 0.32% in 2018 to 0.65% in 2022, *p* < 0.001. In-hospital mortality (IHM) increased over time, but the differences were not significant (6.23% in 2018 vs. 6.79% in 2022). Patients with COPD and RSV infection had, compared with those without RSV infection, a higher use of mechanical ventilation, both invasive (3.44% vs. 1.34%, *p* < 0.001) and noninvasive (8.09% vs. 4.51%, *p* < 0.001) and a higher proportion of intensive care unit (ICU) admission (7.21% vs. 3.9%, *p* < 0.001). After multivariate adjustment, a significant increase in IHM was found from 2018 to 2022 in subjects with and without RSV infection. The presence of RSV infection was associated with a higher mortality (OR 1.22; 95% CI 1.01–1.46). Conclusions: The proportion of RSV infection among patients admitted for COPD increased significantly over time. Patients with COPD and RSV infection had, compared with those without RSV infection, a higher severity, a higher use of mechanical ventilation, and a higher proportion of ICU admission. The presence of RSV infection was associated with IHM. These results can help to identify patients at higher risk and make decisions to avoid the increased risk of hospitalization and mortality in this population.

## 1. Introduction

Respiratory syncytial virus (RSV) infection is a common cause of hospital admission [1,2,3]. Although infection with this virus in adult subjects was described shortly after its identification, its impact on this age group was not established until the widespread use of virus detection techniques with rapid polymerase chain reaction (PCR) tests, which allow monitoring of prevalence. RSV can affect people of any age, but severe disease occurs mainly in the elderly and in those with comorbidities and/or immunosuppression [1,2,3]. In this sense, it has been detected that patients over 60 years of age tend to have more severe respiratory symptoms, which entail a higher risk of developing pneumonia during the course of the disease and/or hospitalization. Among other factors, immunosenescence plays an important role in this relationship [4,5].

A study conducted in the United States between 2017 and 2020 found an annual incidence of RSV infection in adults over 18 years of age between 44.2 and 58.9 per 100,000 inhabitants. The authors recorded a higher incidence of hospitalization in age groups over 65 years of age, being even higher among those over 85 years of age. In relation to associated comorbidities, chronic obstructive pulmonary disease (COPD) stood out, with the hospitalization rate in these patients being between 3.2 and 13.4 times higher than that described for those with RSV infection without COPD [6]. In another study conducted in two European countries, Denmark and Scotland, a higher rate of hospitalization for RSV was found in the age group between 60 and 80 years with comorbidities, being even higher in patients over 85 years of age, especially in patients with respiratory diseases. In the case of COPD or asthma, the rates were up to six times higher in patients over 75 years of age [7].

Viral infection appears to be responsible for up to 50% of COPD exacerbations [8]. The association between COPD exacerbations and RSV infection is not well studied. Thus, some studies have not found a greater severity of this infection in patients with COPD, probably due to the type of population analyzed, characterized by regular exposure to children and early medical care [9]. In contrast, in other studies, the population was recruited among hospitalized patients, which implied a greater severity and fragility of the population with COPD [6,7]. In any case, the incidence and mortality associated with RSV infection in adults have been increasing over time [10,11]. On the other hand, it is estimated that annual mortality from this cause is high [12].

In recent years, there have been substantial advances in the development of new vaccines and immunoprophylactic therapies against RSV [13]. In October 2022, the first long-acting monoclonal antibody against RSV, nirsevimab, was developed. Spain was one of the first countries in Europe to introduce a vaccination program for the prevention of RSV in infants, recommending the administration of nirsevimab to infants under 6 months of age at the beginning or during the 2023–24 RSV season [14]. In addition, in adults, there is a vaccine against RSV that was recently introduced and has demonstrated an overall efficacy, over two seasons, of 67.2%, with an efficacy of 78.8% against severe disease and 73.8% in people with at least one cardiorespiratory comorbidity, such as COPD or heart failure [15]. Thus, vaccination of children and adults may have brought about a change in the epidemiological pattern of RSV infection. Database analysis may be useful for monitoring changes that have occurred over time.

The primary objective of our study was to describe the hospitalizations of subjects with COPD and RSV infection in Spain between 2018 and 2022. Specifically, we analyzed these hospitalizations by age, sex, comorbidities, use of mechanical ventilation, intensive care unit (ICU) admission, in-hospital mortality (IHM), length of hospital stay (LOHS), and costs. Additionally, we compared the characteristics and outcomes of hospitalization in subjects with COPD and RSV infection to those of subjects matched by year of admission, age, and sex who had COPD but without RSV infection. Finally, we identified the variables associated with IHM in subjects with COPD, depending on the presence of RSV.

## 2. Materials and Methods

We have conducted a matched case-control study using data collected from the Spanish public hospitals from 1 January, 2018 to 31 December, 2022 and recorded in the Spanish Hospital Discharge Database (SHDD). The 10th Revision of the International Classification of Diseases, (ICD-10) is used by this database for coding diagnosis (1 to 20) and diagnostic and therapeutic procedures (0 to 20) conducted during the hospitalization. Other outcomes such as admission to ICU, reason for discharge (recovery or death), LOHS, and costs are also recorded in the SHDD [16].

### 2.1. Participants

All subjects hospitalized in public hospitals in Spain between 2018 and 2022 with a COPD code in any of the 20 diagnosis fields of the SHDD were selected (N = 1,792,168). Subjects under 40 years of age were excluded due to the low prevalence of this disease in Spain below that age [17] (N = 271,677), as well as those without recorded age, sex, admission or discharge dates, or reason for discharge (N = 91,203). The COPD population that met the selection criteria was subdivided based on the presence of an ICD-10 code for RSV infection in any diagnostic field (N = 5673 and N = 1,423,615).

To obtain comparable populations, for each subject with COPD and RSV infection, a subject without an RSV code was selected, with the COPD code in the same diagnostic position (1 to 20), as well as the same year of admission, sex, and age. If more than one subject without RSV infection met all the specified conditions, the selection was made randomly. The flowchart of the selection process can be seen in Figure S1.

### 2.2. Study Variables

The primary outcome variable was IHM, defined as the percentage of subjects who died during hospitalization. We also analyzed the evolution of the proportion that required ICU admission, mechanical ventilation (invasive or non-invasive), LOHS, and costs in subjects with and without RSV infection. The covariates described and analyzed included age, sex, comorbidities, and therapeutic procedures. Comorbidity was quantified using the Charlson Comorbidity Index (CCI) following the recommendations of other authors for its calculation in administrative databases coded with ICD10 [18,19]. In addition to the CCI, the presence of depression, asthma, emphysema, bronchiectasis, acute bronchitis, bronchiolitis, influenza, COVID-19, pneumonia, obesity, and obstructive sleep apnea (OSA) was identified. The therapeutic procedures analyzed included the use of invasive and non-invasive mechanical ventilation, long-term (current) use of steroids, and dependence on supplemental oxygen. The ICD10 codes used in this study to define clinical conditions and procedures are shown in Table S1.

### 2.3. Statistical Analysis

To describe the study population according to covariables, we provided absolute number (N) with percentages and means or medians (with standard deviations [SD] or interquartile ranges [IQR]) for categorical and continuous variables respectively. The time trend of categorical variables was evaluated with Cochran–Mantel–Haenszel or Cochran–Armitage tests and for continuous variables with linear regression *t*-tests or the Jonckheere–Terpstra test as adequate. Fisher’s exact test and *t*-tests or the Mann–Whitney test were applied for bivariate analysis for categorical and continuous variables respectively. The Kolmogorov–Smirnov test was used to assess the normal distribution of continuous variables.

Using IHM as the dependent variable, three multivariable logistic regression models were constructed: one for subjects with COPD and RSV infection, another for COPD without RSV infection, and, finally, one for all subjects with COPD to assess the effect of RSV infection on mortality after controlling for confounding factors. The logistic regression multivariable models were built using the “enter modeling” method of STATA 14.0. To construct these models, we followed the recommendations of Hosmer DW and Lemeshow [20]. The process included:

1. Bivariate analysis of each independent variable with the IHM to identify what to include in the multivariable models; 2. We included all the independent variables whose bivariate test was significant (*p* < 0.1) and those we considered scientifically relevant as they may be confounding factors, according to the references reviewed; 3. To fit the multivariable model, the importance of each independent variable was verified. To do this, the Wald statistics for each variable were calculated and compared to each estimated coefficient, with the coefficient from the bivariate model containing only that variable. Based on these criteria, those variables that do not contribute to the model were deleted, and a new model was fitted. The Likelihood Ratio test was applied to compare the new model and the previous model. This process was repeated until only the important independent variables remained in the model. 4. Once the final model was obtained, we assessed the presence of collinearity and checked for two-way interactions between the covariables.

We provided the measure of association odds ratios (OR) with their corresponding 95% confidence intervals (CI).

Stata version 14 (Stata, College Station, TX, USA) was used for all statistical analyses, with a *p*-value of <0.05 (two-tailed) considered significant.

### 2.4. Ethical Considerations

The SHDD is owned by the Spanish Ministry of Health (SMH), which provides it free of charge to any researcher who completes the online application form (6). The SMH is responsible for evaluating and deciding whether requests are scientifically relevant and ethically appropriate. As the SHDD is an administrative and anonymized database, Spanish legislation does not require prior evaluation by an ethics committee or informed consent from participants for conducting epidemiological research [16,21].

## 3. Results

Between 2018 and 2022, a total of 1,429,288 subjects aged 40 years or older with a COPD code were admitted to public hospitals in Spain. Among these patients, 5673 had a recorded diagnosis of RSV infection.

### 3.1. Temporal Evolution in the Number of Hospitalizations with COPD and RSV Infection and Their Characteristics

As shown in Table 1, the number of hospitalizations with COPD and RSV infection increased from 2018 to 2019 (979 to 1352), slightly decreased in 2020 (1024), sharply declined to only 344 in 2021, and returned to higher numbers in 2022 (1944). The proportion of RSV infection among hospitalized COPD patients increased significantly from 0.32% in 2018 to 0.65% in 2022 (*p* < 0.001). Male patients were overrepresented in all years studied (>60%), although the proportion of women appeared to increase in recent years. The mean age remained stable between 2018 and 2022, around 74 years. However, the mean CCI value increased from 1.54 in 2018 to 1.66 in 2022 (*p* = 0.027). ICU admission decreased steadily throughout the study period, from 9.09% in 2018 to 4.89% in 2022 (*p* < 0.001). However, IHM increased but did not show a significant temporal trend (6.23% in 2018 vs. 6.79% in 2022), with a notably higher figure observed in 2020 (9.07%). The median LOHS decreased significantly from 7 days in 2018 to 6 days in 2022. Costs did not vary significantly between 2016 and 2022, with a slight increase observed in 2022, like the trend in IHM.

### 3.2. Hospitalization Characteristics, Outcomes, and Comorbidities in COPD Patients Hospitalized with RSV Infection and Matched COPD Patients Without RSV Infection

Table 2 shows the main characteristics of hospitalizations for COPD and RSV infection compared to those of age- and sex-matched subjects without RSV infection. After matching, we observed that the mean CCI was significantly higher in subjects without RSV infection (1.88 vs. 1.66; *p* < 0.001), as was dependence on supplemental oxygen (17.09% vs. 15.06%; *p* = 0.003). However, the use of invasive mechanical ventilation (3.44% vs. 1.34%; *p* < 0.001) and non-invasive mechanical ventilation (8.09% vs. 4.51%; *p* < 0.001), ICU admission (7.21% vs. 3.9%; *p* < 0.001), and LOHS (7 vs. 6 days; *p* < 0.001) were higher in subjects with RSV infection. IHM was around 7% in both groups of patients (*p* = 0.884), with no difference in the average cost of hospitalization.

When comparing comorbidities between COPD patients hospitalized with and without RSVI, the results shown in Table 3 were obtained. Among chronic diseases, diabetes (27.67% vs. 28.6%; *p* = 0.268), congestive heart failure (23.28% vs. 24.07%; *p* = 0.320), and chronic renal disease (17.92% vs. 17.81%; *p* = 0.883) were the most prevalent. Among respiratory diseases, acute bronchitis was recorded in 16.36% of cases with RSV infection compared to only 4.73% without RSV infection (*p* < 0.001), and influenza (3.4% vs. 2.2%; *p* < 0.001), asthma, and bronchiolitis were also slightly more frequent. However, diagnoses of COVID-19 (1.41% vs. 4.3%; *p* < 0.001) and pneumonia (8.27% vs. 13.07%; *p* < 0.001) were significantly less common in the RSV infection group.

### 3.3. Multivariable Analysis to Identify Factors Associated with IHM in COPD Patients Based on the Presence of RSV Infection

As can be seen in Table 4, after multivariable adjustment, the three populations that were analyzed were those in the higher age groups or who had concomitant comorbidities COVID-19, congestive heart failure, chronic renal disease, myocardial infarction, cancer, or cerebrovascular disease with a significant and positive OR for IHM. Invasive and non-invasive mechanical ventilation, as well as dependence on supplemental oxygen, were procedures associated with a higher IHM.

A significant increase in IHM from 2018 to 2022 was found in both subjects with RSV infection and those without this diagnosis. Finally, in the total study population, after adjusting for the confounding effect of the remaining variables, the presence of RSV infection was associated with a slightly but significantly higher IHM (OR 1.22; 95% CI 1.01–1.46).

## 4. Discussion

In this study, conducted at a national level, we found that the number of admissions for RSV infection in patients with COPD increased from 2018 to 2019, decreased slightly in 2020, fell sharply in 2021, and returned to higher figures in 2022. These changes can be explained by the characteristics of RSV transmission and are influenced by the protective measures adopted in the face of the COVID-19 pandemic. As it is a seasonal virus that occurs mainly from October to March, its transmission was affected from March 2020 onwards, which would explain why a sharper drop is not reflected in 2020, since admissions from January to March 2020 were not influenced by the pandemic. The measures applied to prevent the transmission of SARS-CoV-2 also prevented that of RSV. The use of masks, hand hygiene, and social distancing imposed on the population in Spain from 2020–2021 may explain the sharp drop in the number of COPD and RSV infection admissions observed in 2021 [22]. A review published in 2023 found no significant differences between mask wearers in relation to the spread of viral respiratory infections and found only low evidence regarding hand hygiene [23]. Therefore, the results obtained can be attributed to the lack of exposure to the virus due to social distancing [24,25]. Following the end of the lockdown, social distancing and mask-wearing measures and compliance decreased in the Spanish population, possibly leading to an increase in RSV transmission once again.

In any case, the significant increase in the proportion of RSV infection cases in patients hospitalized for COPD from 2018 to 2022 could be attributed to the increasing number of respiratory virus detections. Thus, after the COVID-19 pandemic, the use of multiplex rapid PCR tests for the detection of respiratory viruses, including mainly influenza A and B viruses, RSV, and SARS-CoV-2, has become widespread in hospitals [26].

This study clearly shows that the prevalence of COPD was substantially higher in men than in women, which might be due to their higher smoking rate, although we do not have information on this. In addition, subgroup analysis also showed that men were more prone to RSV infection than women. Previous studies have shown sex differences in the incidence and severity of respiratory tract infections. Specifically, in RSV infections, boys have been found to be more severely affected than girls [27]. These findings indicate potential sex-related differences in susceptibility or exposure to certain respiratory viruses during specific age group [28].

ICU admissions decreased steadily throughout the study period, which can be justified by the same reason, since the widespread use of PCR would increase the total number of cases detected, most of them less severe. The massive international use of virus detection techniques during the COVID-19 pandemic, between 2020 and 2022, caused an increase in incidence due to underdiagnosis in previous years. This fact has allowed us to better understand the epidemiology of RSV infection, which has been associated with an improvement in clinical practice and the development of new therapeutic and vaccination strategies [29]. It should be noted that the active search for respiratory viruses as a cause of respiratory infections is essential to avoid the overtreatment of these patients with antibiotic therapy.

When comparing patients with COPD and RSV infection with those with COPD without RSV infection in our study, we found a greater severity in the first group. Thus, patients with COPD and RSV infection required a higher percentage of admission to the ICU and the use of mechanical ventilation, both invasive and noninvasive, also presenting a higher LOHS. In a study published in 2020 in which hospitalizations for RSV infection were analyzed between 2012 and 2015, it was found that the rates of hospitalization for RSV were 10 times higher in patients with COPD, even segregating into two age groups (50 to 64 years and 65 to 80 years) [30]. In another study conducted in the USA between July 2022 and June 2023, in which 756 high-risk patients were analyzed, 33.7% with COPD, a higher proportion of severe disease, was also found in this subgroup of patients [31]. In another American series of 1795 patients with RSV infection, recruited between 2011 and 2015, it was described that 44.2% of them were hospitalized and, of these, 95% were considered high risk. This group included 53.7% of patients with COPD, with the remainder presenting other comorbidities (history of pneumonia, heart failure, or immunosuppression). The average hospital stay was 7 days in high-risk patients, compared with 5.5 days in the remaining patients, and the in-hospital mortality rate was 4.2% versus 0%, respectively [32]. Furthermore, in an Israeli series of patients hospitalized between 2016 and 2022, the presence of lung disease was also found to be a risk factor for RSV hospitalization [33]. In another study conducted in Denmark and Scotland between 2010 and 2016, it was concluded that patients over 45 years of age with comorbidities had a higher risk of contracting a serious RSV infection, finding that patients with COPD have an increased risk of hospitalization due to RSV infection, between two and four times higher. Analyzing the age groups, they found that hospitalization rates increased with increasing age, mainly in patients with COPD and asthma, showing rates more than six times higher in patients over 75 years of age compared to the general population [7].

COPD patients and RSV infection in our study had a higher frequency of acute bronchitis, influenza virus infection, and bronchiolitis compared to those with COPD without RSV infection. A European study conducted between 2006 and 2018 in hospitalized patients with RVS infection found higher rates than ours, with 10% of bronchitis and bronchiolitis in Denmark and 6% of influenza virus co-infection in Scotland and Denmark [34].

It is surprising that the proportion of patients with COPD and RSV infection diagnosed with SARS-CoV-2 infection and pneumonia in our study was significantly lower than those with COPD without RSV infection. Viral co-infections are present in confirmed cases of COVID-19 and include most respiratory viruses, among which RSV is found. Although there are no large series, the prevalence of viral co-infection is low, although this fact is associated with greater disease severity. Landsbury et al. found that RSV was the main virus diagnosed in patients with SARS-CoV-2 infection [35]. Subsequently, different publications have studied the prevalence, severity, and mortality rates of viral infections, including SARS-CoV-2 and RSV infections, but the results are not comparable since the immune status of the population has been changing over the years based on exposure and vaccination. Thus, we can observe the influence of vaccination in an American study conducted on patients hospitalized for respiratory infection between 2022 and 2023. These authors found less severe disease in the group of patients with RSV, COVID-19, and influenza infections who were vaccinated compared to those who were not vaccinated. Among the viruses analyzed, RSV infection was associated with the presence of more severe respiratory disease [36]. Another study analyzed SARS-CoV-2 co-infection with other viral infections, finding 9.9% of cases, 16.7% of which were caused by RSV. In the group of patients over 65 years of age with viral co-infection, a longer hospital stay and a higher probability of fatal outcome were observed compared to SARS-CoV-2 infection alone, although the study was limited by the small number of co-infected patients [37]. In our study, we found 1.41% co-infection in patients with COPD.

Regarding the development of pneumonia in patients with COPD, fewer cases were found in our series than in patients with COPD without RSV infection. In the EPIC (Etiology of Pneumonia in the Community) study conducted in the United States in the period from 2010–2012, the presence of the virus was detected in 26% of adults hospitalized with community-acquired pneumonia, this figure being 73% in the case of children [38]. A review published in 2019 suggested that virus detection techniques have limitations and that the role of viruses in pneumonia could be greater since nasopharyngeal exudate samples may be negative in the case of pneumonia of viral etiology [39].

In our study, we found higher in-hospital mortality associated with advanced age, congestive heart failure, myocardial infarction, chronic kidney disease, cerebrovascular disease, cancer, and COVID-19 infection. We found no differences between COPD patients with and without RSV infection. Different authors have found the same association [40]. Thus, Njue et al. found a similar association between age and comorbidities, highlighting the importance of immunosenescence [40]. Throughout the period of our analysis, we found a significant increase in IHM from 2018 to 2022, both in the population with RSV infection and in those without such infection, a fact probably attributable to the widespread use of multiple rapid PCR tests for the detection of respiratory viruses, which would reduce underdiagnosis [26].

In our series, we found no differences in IHM between the COPD population with RSV infection and those without it, which was 7.11 and 7.04%, respectively, although after adjustment for the confounding effect of the variables, the presence of RSV was associated with a slight increase in in-hospital mortality. Ackerson et al. studied mortality associated with RSV versus influenza without finding differences in IHM [41]. Celante et al. found a mortality associated with RSV infection of 6.6% [42], and another Israeli series found mortality associated with RSV infection at 6.2% [33].

The main strength of our study lies in the high number of participants analyzed and the long analysis period, in addition to the exhaustive data collection from the Spanish National Database of Hospital Discharges, which includes information from practically all Spanish public hospitals. However, our study also has limitations, mainly derived from the administrative data source. Therefore, definitions depend on the presence of a precise and relevant diagnosis or procedure code, and diagnosis codes may not accurately distinguish disease severity. Another limitation of this study is that we did not have laboratory or drug administration data. Furthermore, we only had hospitalization data, not follow-up after discharge.

In our study population, there are many patients without RSV for each patient with RSV, so it would be possible to use a larger than the selected 1:1 case-control ratio. However, according to several authors, the most optimum case-to-control ratio is 1:1 as the chi-square test for independence is most powerful if the number of cases is the same as the number of controls [43,44]. However, if there are a limited number of cases, we can increase the number of controls to increase the statistical power of the study [45,46]. In our study, we had 5673 cases. When we used the methods described by Sinha S and Mukherjee B, we confirmed that with our sample size, a case-to-control ratio is 1:1, would be adequate for an estimated OR of 1.1. or over [46]. Therefore, with our sample size, a ratio of 1:1 already provides adequate statistical power, and larger ratios (2:1, 3:1, etc.) would provide no benefit in the precision of the estimates [45,46].

Finally, we were surprised by the difference between the results of the bivariate and multivariate analysis regarding the association of the presence of RSV infection with the IHM. The difference is probably due to differences in the types of comorbidities determining higher mortality among RVS patients after multivariable adjustment. Therefore, this result should be interpreted with caution and will have to be verified in studies with more detailed clinical data that should include, among others, the type, time of evolution, and severity of the underlying comorbidities among COPD patients.

## 5. Conclusions

The proportion of RSV infection among patients admitted for COPD increased significantly over time. Patients with COPD and RSV infection had, compared with those without RSV infection, a higher severity, a higher use of mechanical ventilation, and a higher proportion of ICU admission. The presence of RSV infection was associated with higher mortality. These results can help to identify patients at higher risk and make decisions to avoid the increased risk of hospitalization and mortality in this population.

## Figures and Tables

**Table 1:** Evolution over time in the number and characteristics of patients hospitalized with a diagnosis of chronic obstructive respiratory disease (COPD) and respiratory syncytial virus (RSV) infection in Spain (2018–2022).

		2018	2019	2020	2021	2022	*p* Trend
Number of patients with COPD, n		304,991	303,319	256,365	264,557	300,056	NA
Number of patients with COPD and RSV infection, n (%)		979 (0.32)	1352 (0.45)	1054(0.41)	344 (0.13)	1944 (0.65)	<0.001
Sex n (%)	Men	657 (67.11)	940 (69.53)	749 (71.06)	207 (60.17)	1272 (65.43)	<0.001
	Women	322 (32.89)	412 (30.47)	305 (28.94)	137 (39.83)	672 (34.57)	
Age, mean (SD)		74.18 (11.23)	75.19 (11.01)	75.12 (10.91)	74.46 (12.12)	74.59 (11.24)	0.177
Age groups, n (%)	40–64 years	318 (32.48)	401 (29.66)	301 (28.56)	123 (35.76)	642 (33.02)	0.024
	64–74 years	305 (31.15)	416 (30.77)	365 (34.63)	91 (26.45)	603 (31.02)	
	75+ years	356 (36.36)	535 (39.57)	388 (36.81)	130 (37.79)	699 (35.96)	
CCI, n (%)	0	344 (35.14)	444 (32.84)	327 (31.02)	119 (34.59)	661 (34)	0.339
	1–2	403 (41.16)	568 (42.01)	429 (40.7)	132 (38.37)	796 (40.95)	
	≥3	232 (23.7)	340 (25.15)	298 (28.27)	93 (27.03)	487 (25.05)	
CCI, mean (SD)		1.54 (1.69)	1.68 (1.82)	1.8 (1.94)	1.77 (2.09)	1.66 (1.87)	0.027
Admission to ICU, n (%)		89 (9.09)	118 (8.73)	93 (8.82)	14 (4.07)	95 (4.89)	<0.001
IHM, n (%)		61 (6.23)	93 (6.88)	95 (9.01)	22 (6.4)	132 (6.79)	0.107
LOHS, median (IQR)		7 (8)	7 (7)	8 (7)	7 (6)	6 (6)	<0.001
Costs in euros, mean (SD)		4339 (5925)	4499 (5709)	5039 (5944)	4234 (3471)	4346 (4230)	0.055

CCI: Charlson comorbidity index. SD: Standard deviation. ICU: Intensive care unit. IHM: In-hospital mortality. IQR: Inter quartile range. LOHS: Length of hospital stay.

**Table 2:** Characteristics of patients hospitalized with a diagnosis of chronic obstructive respiratory disease and respiratory syncytial virus (RSV) infection and age-sex matched patients without RSV infection in Spain (2018–2022).

		RSV Infection	No RSV Infection	*p*
Sex n (%)	Men	3824 (67.43)	3824 (67.43)	NA
	Women	1847 (32.57)	1847 (32.57)	
Age, mean (SD)		74.74 (11.17)	74.74 (11.17)	NA
Age groups, n (%)	40–64 years	1785 (31.48)	1785 (31.48)	NA
	64–74 years	1780 (31.39)	1780 (31.39)	
	75+ years	2106 (37.14)	2106 (37.14)	
CCI, mean (SD)		1.68 (1.86)	1.88 (2.01)	<0.001
CCI, n (%)	0	1895 (33.42)	1697 (29.92)	<0.001
	1–2	2328 (41.05)	2340 (41.26)	
	3+	1448 (25.53)	1634 (28.81)	
Invasive mechanical ventilation, n (%)	Yes	195 (3.44)	76 (1.34)	<0.001
Non-invasive mechanical ventilation n (%)	Yes	459 (8.09)	256 (4.51)	<0.001
Long-term (current) use of steroids, n (%)	Yes	289 (5.1)	262 (4.62)	0.238
Dependence on supplemental oxygen, n (%)	Yes	854 (15.06)	969 (17.09)	0.003
Admission to ICU, n (%)	Yes	409 (7.21)	221 (3.9)	<0.001
IHM, n (%)	Yes	403 (7.11)	399 (7.04)	0.884
LOHS, median (IQR)		7 (7)	6 (7)	<0.001
Costs in euros, mean (SD)		4503 (5226)	4483 (4799)	0.830

CCI: Charlson comorbidity index. SD: Standard deviation. ICU: Intensive care unit. IHM: In-hospital mortality. IQR: Inter quartile range. LOHS: Length of hospital stay. NA: Not available.

**Table 3:** Comorbidities of patients hospitalized with a diagnosis of chronic obstructive respiratory disease and respiratory syncytial virus (RSV) infection and age-sex matched controls without RSV infection in Spain (2018–2022).

	RSV Infection	No RSV Infection	*p*
Congestive heart failure, n (%)	1320 (23.28)	1365 (24.07)	0.320
Myocardial infarction, n (%)	44 (0.78)	65 (1.15)	0.043
Chronic renal disease, n (%)	1016 (17.92)	1010 (17.81)	0.883
Depression, n (%)	251 (4.43)	260 (4.58)	0.684
Diabetes, n (%)	1569 (27.67)	1622 (28.6)	0.268
Liver disease, n (%)	260 (4.58)	288 (5.08)	0.220
Peripheral vascular disease, n (%)	452 (7.97)	522 (9.2)	0.019
Cerebrovascular disease, n (%)	211 (3.72)	342 (6.03)	<0.001
Cancer, n (%)	542 (9.56)	798 (14.07)	<0.001
Asthma, n (%)	317 (5.59)	263 (4.64)	0.021
Emphysema, n (%)	912 (16.08)	927 (16.35)	0.702
Bronchiectasis, n (%)	184 (3.24)	175 (3.09)	0.629
Acute bronchitis, n (%)	928 (16.36)	268 (4.73)	<0.001
Bronchiolitis, n (%)	135 (2.38)	5 (0.09)	<0.001
Influenza, n (%)	193 (3.4)	125 (2.2)	<0.001
COVID-19, n (%)	80 (1.41)	244 (4.3)	<0.001
Pneumonia, n (%)	469 (8.27)	741 (13.07)	<0.001
Obesity, n (%)	823 (14.51)	820 (14.46)	0.936
OSA, n (%)	778 (13.72)	787 (13.88)	0.806

OSA: Obstructive sleep apnea.

**Table 4:** Factors associated with hospital mortality among patients hospitalized with a diagnosis of chronic obstructive respiratory disease according to the presence of respiratory syncytial virus (RSV) infection in Spain (2018–2022).

		RSV Infection OR (95% CI)	No RSV Infection OR (95% CI)	All Patients OR (95% CI)
Age groups	40–64 years	1	1	1
	64–74 years	1.8 (1.3–2.49)	1.35 (0.98–1.86)	1.55 (1.24–1.94)
	75+ years	3.34 (2.42–4.61)	2.82 (2.09–3.8)	3.06 (2.46–3.81)
Congestive heart failure	Yes	1.3 (1.02–1.65)	1.34 (1.06–1.7)	1.32 (1.11–1.56)
Myocardial infarction	Yes	3.39 (1.57–7.33)	1.43 (0.65–3.15)	2.16 (1.26–3.7)
Chronic renal disease	Yes	1.71 (1.33–2.2)	1.53 (1.19–1.98)	1.59 (1.33–1.91)
Cerebrovascular disease	Yes	1.53 (0.96–2.45)	1.67 (1.16–2.41)	1.64 (1.23–2.18)
Cancer	Yes	2.1 (1.54–2.87)	3.25 (2.55–4.15)	2.76 (2.29–3.34)
COVID-19	Yes	1.72 (0.85–3.50)	2.16 (1.44–3.24)	1.94 (1.37–2.74)
Invasive mechanical ventilation	Yes	3.27 (2.42–4.43)	1.33 (0.83–2.15)	2.4 (1.87–3.08)
Non-invasive mechanical ventilation	Yes	2.53 (1.54–4.15)	3.39 (1.81–6.35)	2.8 (1.91–4.11)
Dependence on supplemental oxygen	Yes	1.69 (1.29–2.2)	1.42 (1.09–1.84)	1.51 (1.25–1.82)
Year		1.09 (1.03–1.19)	1.07 (1.01–1.15)	1.08 (1.02–1.12)
Respiratory syncytial virus	Yes	NA	NA	1.22 (1.01–1.46)

NA: Not available.

## Data Availability

According to the contract signed with the Spanish Ministry of Health and Social Services, which provided access to the databases from the Spanish National Hospital Database, we cannot share the databases with any other investigator, and we have to destroy the databases once the investigation has concluded. Consequently, we cannot upload the databases to any public repository. However, any investigator can apply for access to the databases by filling out the questionnaire available at https://www.sanidad.gob.es/estadEstudios/estadisticas/estadisticas/estMinisterio/SolicitudCMBD.htm (accessed on 16 November 2024). All other relevant data are included in the paper.

## Supplementary Materials

The following supporting information can be downloaded at: https://www.mdpi.com/article/10.3390/diseases13010023/s1, Table S1: International Classification of Diseases 10th Revision (ICD10) codes used in this investigation; Figure S1: Flowchart of COPD patient’s selection and hospital outcome according to the presence of respiratory syncytial virus infection.
