# Supplementary material for: Prevalence of Respiratory Syncytial Virus Infection in Hospitalized COPD Patients in Spain Between 2018–2022
Source: Diseases. 2025 Jan 20;13(1):23. doi: 10.3390/diseases13010023 (PMC11764113; doi:10.3390/diseases13010023)

Table S1. International Classification of Diseases 10th Revision (ICD10) codes used in this investigation.

Diagnosis or procedure	IC10 codes
Respiratory syncytial virus infection	J12.1, J20.5, J21.0 , B97.4
COPD	J41, J42, J43, J44
Congestive heart failure	I50
Myocardial infarction	I21, I22
Chronic renal disease	N18
Depression	F32
Diabetes	E10, E11
Liver disease	K72 to K77
Peripheral vascular disease	I71, I790, I739, R02, Z958, Z959
Cerebrovascular disease	I60, I61, I62, I63, I65, I66,G450, G451, G452, G458, G459, G46, I64, G454, I670, I671, I672, I674, I675, I676, I677 I678, I679, I681, I682, I688, I69
Cancer	C00, C26, C30, C34, C37, C41, C43, C45., C58, C60, C76, C81, C85, C88, C90, C97, C77, C80
Asthma	J45
Emphysema	J43
Bronchiectasis	J47
Acute bronchitis	J20
Bronchiolitis	J21
Influenza	J09-J11
COVID 19	B97.29 U07.1
Pneumonia	J13 to J18 and J95.851
Obesity	E66.09, E66.1 E66.3, E66.8 E66.9 E66.2, E66.01
Obstructive Sleep Apnea (OSA)	G47.3 toG473.9
Invasive mechanical ventilation	5A1935Z 5A1945Z, 5A1955Z,
Non-invasive mechanical ventilation	5A09357, 5A09457, 5A09557
Code for long term (current) use of steroids	Z79.5
Dependence on supplemental oxygen	Z99.81

Figure S1. Flowchart of COPD patient's selection and hospital outcome according to the presence of respiratory syncytial virus infection

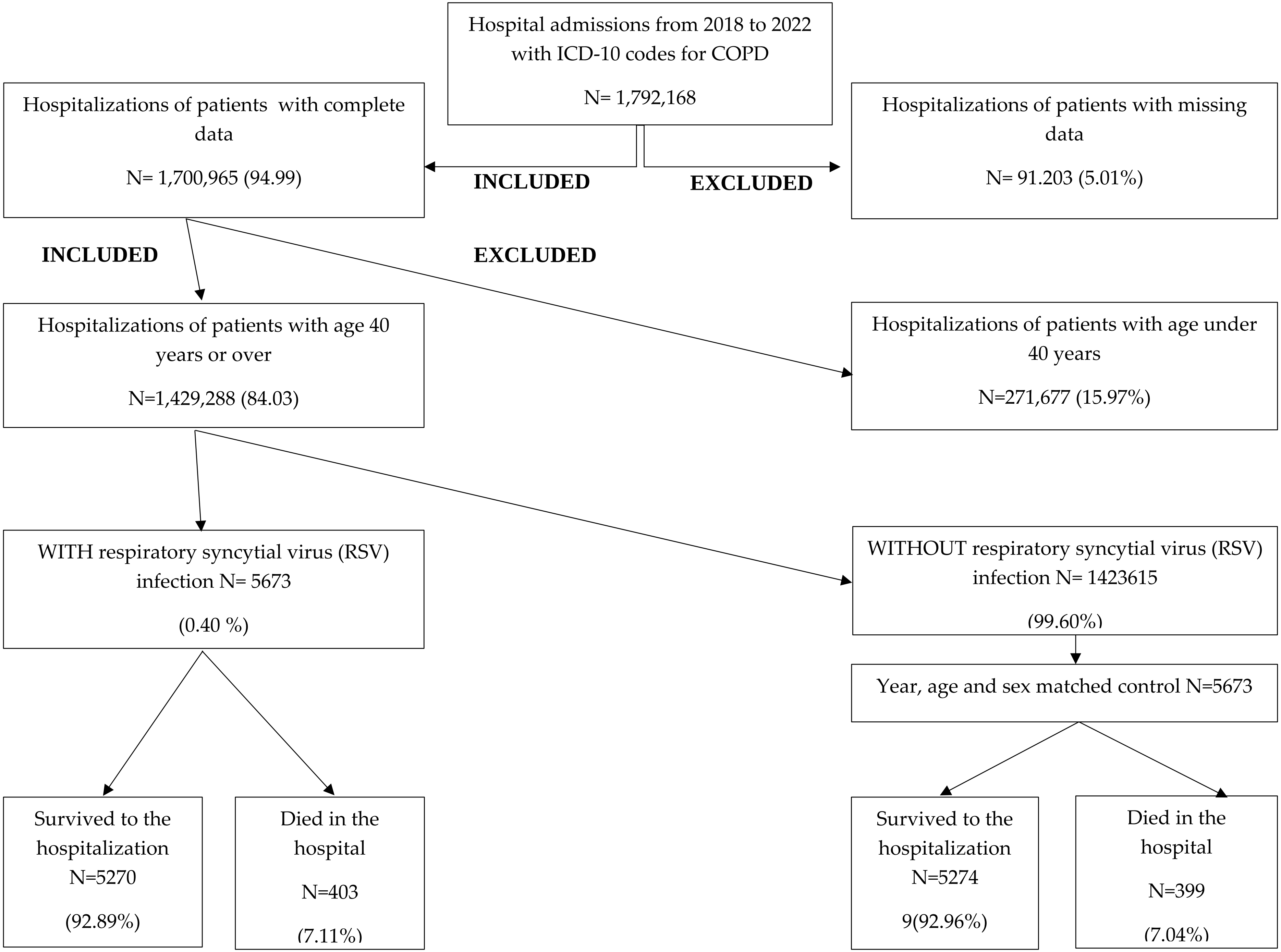

Supplement: Supplementary file 1 [file diseases-13-00023-s001.zip › diseases-3404392-supplementary.pdf]
